# Immune checkpoint blockade enhances chemophototherapy in a syngeneic pancreatic tumor model

**DOI:** 10.1063/5.0099811

**Published:** 2022-09-23

**Authors:** Sanjana Ghosh, Xuedan He, Wei-Chiao Huang, Jonathan F. Lovell

**Affiliations:** Department of Biomedical Engineering, University at Buffalo, State University of New York, Buffalo, New York 14260, USA

## Abstract

Pancreatic cancer (PaCa) suffers from poor treatment options for locally advanced cases. Chemophototherapy (CPT) is an emerging anti-tumor modality, and porphyrin–phospholipid liposomes have been shown to be versatile drug carriers for CPT in preclinical rodent models. Here we show that in the syngeneic subcutaneous KPC PaCa tumor model, exhausted CD8^+^ T cells are localized in the tumor, and that CPT is enhanced in combination with immune checkpoint blockade (ICB). Addition of ICB using anti-programmed cell death 1 (PD-1) and cytotoxic T-lymphocyte-associated protein 4 (CTLA-4) antibodies resulted in ablation of medium-sized, established KPC tumors (∼200 mm^3^) without recurrence for over 100 days. Mice rejected subsequent tumor re-challenge. Flow cytometry and tumor slice analysis following injection of a fluorescently labeled anti-PD-1 antibody showed that CPT improved antibody delivery to the tumor microenvironment. Treatment of large established tumors (∼400 mm^3^) using with CPT and ICB induced appreciable tumor regression and delay in regrowth. Taken together, these data demonstrate the utility of combining CPT with immunotherapies.

## INTRODUCTION

Pancreatic cancer (PaCa) is an aggressive malignancy and the seventh most lethal cancer in the world.^1^ Over 90% of PaCa mortality is due to pancreatic ductal adenocarcinoma (PDAC), which tends to feature hypovascularity and desmoplasia that makes drug delivery challenging.^2,3^ The prognosis for PDAC is poor with a 9% five-year survival rate.^4^ Only about 20% of cases are candidates for initial resection, given the propensity for late diagnosis and that the tumors often impinge on nearby critical vessels.^5^ All these factors make it difficult to control PaCa by the traditional methods of chemotherapy, radiotherapy, or surgical resection.^6^ Chemotherapy remains the conventional treatment for unresectable tumors.^7^

Immunotherapy represents a breakthrough in cancer treatment. Extensive research into the function of immune system in identifying malignant cells has led to an understanding of the regulatory mechanisms that control immune responses during oncogenesis.^8,9^ One approach is the use of immunomodulators that block immune checkpoints such as PD-1 or PD-L1 (programmed cell death 1 or its ligand) and cytotoxic T-lymphocyte-associated protein 4 (CTLA-4).^10^ CTLA-4, also known as CD152 (cluster of differentiation 152), is a transmembrane glycoprotein of the immunoglobulin superfamily, which is important in immune checkpoint blockade (ICB) for T cell activation.^11^ This protein receptor is expressed in humans, primates, cows, sheep, dogs, cats, rats, and mice.^11–14^ The gene responsible for the expression of CTLA-4 is present on chromosome 2 in humans and chromosome 1 in mice.^12,15^ It is typically expressed upon activation of both CD4^+^ and CD8^+^ T cells and is a cell membrane receptor.^16,17^ It is structurally homologous to CD28 (∼31%) and has higher affinity than CD28 for the co-stimulatory molecules B7 expressed on antigen-presenting cells (APCs; typically macrophages, dendritic cells, and B cells) and has attracted tremendous research interest.^18,19^ Ig-family protein CD28 binds to B7 family of immune-regulatory ligands B7–1 and B7–2 on APCs and instigates the production of interleukin-2 (IL-2) and anti-apoptotic factors leading to the proliferation of T cells.^20^ However, increased T cell activity can induce dysregulation of T cells leading to autoimmunity, hypersensitivity, and dysfunctional T cell responses in cancer.^21^ MHC peptide/TCR (major histocompatibility complex, MHC peptide/T cell receptor, TCR) signaling engages a negative feedback loop by recruiting CTLA-4 to downregulate T cell responses.^22^ Another such negative regulatory protein on T cells is PD-1. Like CTLA-4, it is also a cell surface protein of the immunoglobulin superfamily.^23^ Following MHC-peptide/TCR signaling, PD-1 is expressed on various hematopoietic cells including T cells, B cells, natural killer T cells, macrophages, monocytes, and dendritic cells.^24^ It binds to two ligands—programmed cell death ligand 1 (PD-L1, expressed in various hematopoietic and non-hematopoietic cells) and programmed cell death ligand 2 (PD-L2, expressed in APCs and some B cells).^25,26^ Chronic and prolonged exposure to antigens in cancer leads to increased expression of CTLA-4 and PD-1 resulting in acquired tolerance of antigens, thus nullifying effector functions such as cytokine production, cytotoxicity, and T cell proliferation. Thus, a balance of immunostimulatory signaling is essential for continuous and productive immune responses.^27,28^ Research efforts have led to the clinical development of monoclonal antibodies targeting CTLA-4, PD-1, and others. In 2011, the United States Food and Drug Administration (FDA) approved the first ICB: a monoclonal antibody (mAb) targeting CTLA-4. Numerous mAbs that target PD-1 have been approved by the FDA.

Despite success in clinical trials, ICB-based therapeutic endeavors have had limited success in PaCa.^29,30^ This is attributed to its immune evasive nature and hypovascular features that inhibit infiltration of anti-cancer immune cells. Of the mainstay chemotherapy regimens for PDAC, FOLFIRINOX, consisting of Oxaliplatin, leucovorin, irinotecan (IRI), and 5-fluorouracil, is common and Gemcitabine with albumin-bound paclitaxel is also often used.^31^ Another ablation modality that is undergoing evaluation for PaCa is the photodynamic therapy (PDT).^32–35^ Numerous PDT approaches for PaCa have been reported.^36–39^ A nanoliposomal formulation of IRI has been FDA approved for the treatment of late-stage pancreatic cancer.^40,41^ In light of this, we recently developed a photoactivatable liposomal formulation of IRI by incorporating a small (1%) mole fraction of porphyrin–phospholipid (PoP), a photosensitizing agent that stably integrates into a bilayered lipid structure and enables light-triggered drug release.^42^ We refer to the approach of combining chemotherapy with PDT as chemophototherapy (CPT), which has proven to be an effective tumor ablation modality in the preclinical setting.^43^ CPT using PoP liposomes generally results in vascular damage that enhances the delivery of drug-loaded liposomes to the tumor.^44–50^ In this study, we investigate whether photoactivatable IRI coupled with immunotherapy (in the form of ICB) could be beneficial for tumor treatment in an immunocompetent murine PaCa tumor model.

## RESULTS

### IRI-PoP liposomes

IRI-PoP liposomes were produced as recently reported, using an ammonium sucrosulfate remote loading method, resulting in liposomes approximately 100 nm in diameter with good encapsulation stability in serum and storage.^42^ Drug loading was 92% [Fig. S1(A)] and liposomes released encapsulated IRI within a few minutes of irradiation with a 665 nm laser [Fig. S1(B)]. Liposome morphology was spherical with encapsulated IRI within the aqueous core [Fig. S1(C)]. When liposomes were incubated with KPC cells *in vitro*, cellular uptake of IRI was observable, especially with IRI liposomes that had been irradiated to trigger the release of their cargo (Fig. S2). This led to higher cytotoxicity of irradiated IRI liposomes (Fig. S3).

**FIG. 1.**
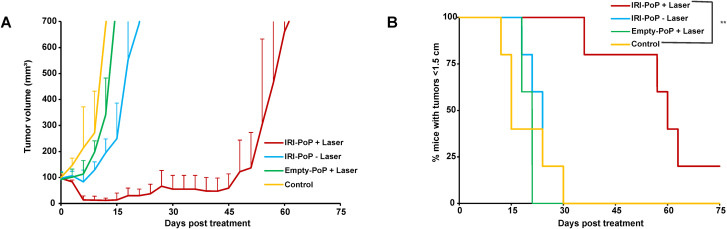
Recurrence of small KPC tumors after CPT with IRI-PoP liposomes. Immunocompetent mice with KPC tumor xenografts (tumor volume ∼100 mm^3^) were treated with IRI-PoP liposomes as indicated with 18 mg/kg IRI dose (2 mg/kg PoP dose) or empty PoP liposomes with equivalent PoP. Mice that received laser treatment were treated using a 665 nm laser with a laser fluence rate of 250 mW/cm^2^ with a total fluence of 250 J/cm^2^ at 1 h DLI. (a) Tumor volume growth and (b) % mice with tumors <1.5 cm in diameter over time. Each data point shows the mean ± SD for n = 5 mice per group. Statistical analysis of Kaplan Meier curve was performed by Log-rank (Mantel-Cox) test, ^*^P <0.05, ^**^P <0.01.

**FIG. 2.**
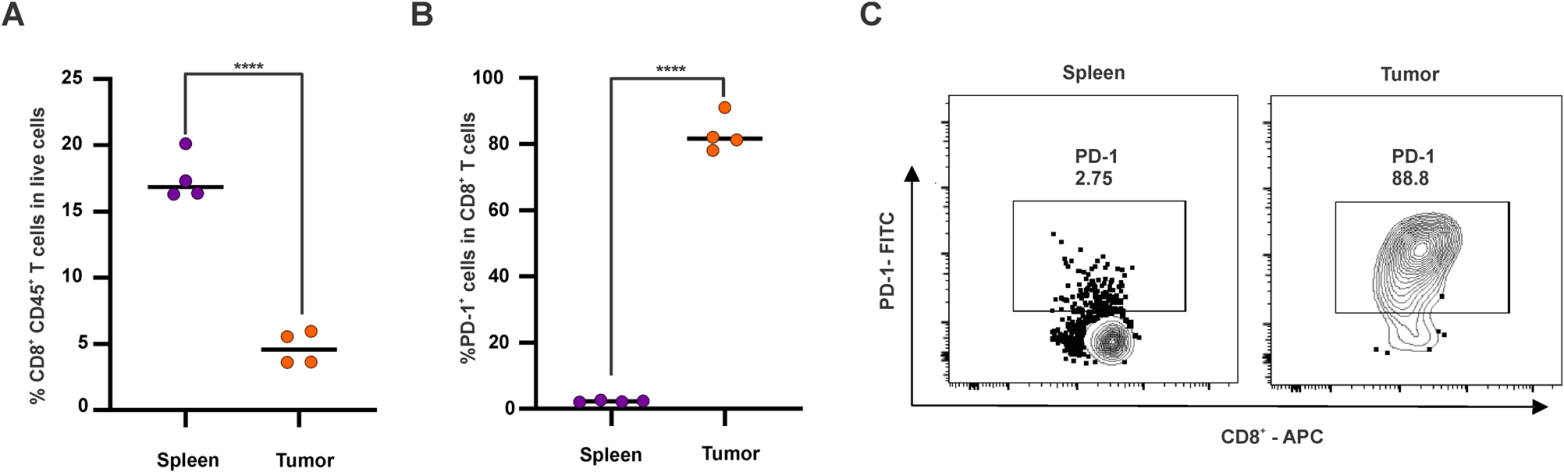
T cell exhaustion in the KPC tumor microenvironment. (a) Percentage of CD8^+^ CD45^+^ T cells in live cells in tumor and spleen. (b) Percentage and (c) flow cytometry gating of PD-1^+^ T cells in CD8^+^ T cell populations in tumor and spleen. Statistical analysis of %CD8^+^ CD45^+^ T cells in live cells (a) and %PD-1^+^ cells in T cells (b) between indicated groups was performed by Student T test, ^*^P <0.05, ^**^P <0.01, ^***^P <0.001, ^****^P <0.0001. Mean ± SD for n = 4 mice per group.

**FIG. 3.**
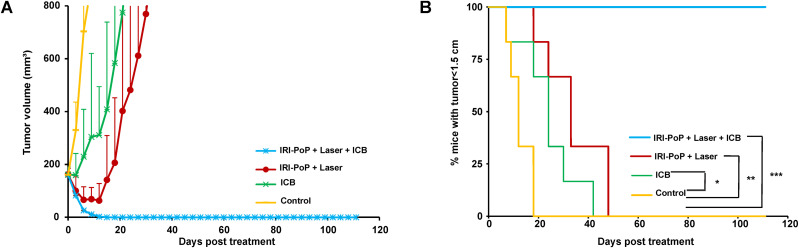
Synergistic chemo-photo-immunotherapy for medium-sized KPC tumor xenografts. Immunocompetent mice with KPC tumor xenografts (∼200 mm^3^) were untreated/treated with IRI-PoP liposomes with 18 mg/kg IRI dose (2 mg/kg PoP dose). Mice that received laser treatment were treated using a 665 nm laser with a laser fluence rate of 250 mW/cm^2^ with a total fluence of 250 J/cm^2^ at 1 h DLI. Mice receiving ICB (5 mg/kg anti-PD-1 and 5 mg/kg anti-CTLA4) were administered intraperitoneally three times starting from the day of laser treatment at an interval of 3 days. (a) Tumor volume growth and (b) % mice tumors <1.5 cm in diameter over time. Mean ± SD for n = 6. Statistical analysis of Kaplan Meier curve was performed by Log-rank (Mantel-Cox) test, ^*^P < 0.05, ^**^P < 0.01, ^***^P < 0.001.

### Treatment of small KPC tumors with CPT using IRI-PoP liposomes

To evaluate the anti-tumor efficacy of IRI-PoP liposomes in murine KPC pancreatic ductal adenocarcinoma, immunocompetent mice implanted with KPC tumors (∼100 mm^3^) were randomly grouped into four cohorts: IRI-PoP + laser, IRI-PoP − laser, empty PoP + laser, and untreated. All mice were untreated/treated intravenously with 18 mg/kg IRI-PoP liposomes or empty-PoP liposomes with equivalent PoP dose (2 mg/kg PoP). The mice that received laser treatment were treated with a laser fluence rate of 250 mW/cm^2^ at fluence of 250 J/cm^2^ 1 h after drug administration, a drug-light-interval (DLI) found to produce more effective responses than longer ones.^53^ The mice that received chemophototherapy showed initial shrinkage of the tumors whereas all mice from other cohorts showed faster tumor growth. However, regrowth of tumor volume was observed in mice that received IRI-PoP + laser by the 18th day from treatment, and eventually, all mice except one reached the end point of the study [Fig. 1(a)]. This indicates that a single treatment with photodynamic IRI-PoP liposomes could not effectively cure even relatively small KPC tumors. All mice that received empty PoP + laser had to be euthanized by 20th day from treatment [Fig. 1(b)]. All other mice quickly reached end point in their tumor growth and they had to be sacrificed by the 30th day from treatment.

### Overexpression, exhaustion, and dysfunction of CD8^+^ T cells in the KPC tumor microenvironment

To examine immunological factors behind the regrowth of KPC tumors, the expression of PD-1, one of the main inhibitory receptors for T cell exhaustion, was tested in both spleen and the KPC tumor microenvironmnent (TME). Although a higher percentage of CD8^+^ CD45^+^ T cells was observed in the spleen as compared to tumors [Fig. 2(a)] based on flow cytometry gating, the expression of PD-1 of intratumoral CD8^+^ T cells was significantly higher compared with splenic CD8^+^ T cells [Figs. 2(b) and 2(c)]. PD-1 regulates immune system by restricting immune-mediated tissue damage via uncontrolled activation of T cells. Conversely, chronic exposure of antigens to tumor-infiltrating lymphocytes (TILs) can cause the expression of PD-1 on T cells leading to blunted immune responses The higher frequency of PD-1 expression of intratumoral CD8^+^ T cells [Figs. 2(b) and 2(c)] suggests potential overstimulation of the TCR by sustained exposure to tumor antigens. The expression of PD-1 can lead to impaired effector functions related to cytokine production, cytotoxicity, and proliferation during T cell dysfunction.^54–57^ A TME containing exhausted CD8^+^ T would facilitate the regrowth of cancer cells, which is potentially a reason behind the tumor recurrence observed earlier (Fig. 1).

### Combined CPT and immunotherapy ablate medium-sized KPC tumors and induce memory immune responses

To investigate the synergistic effect of chemo-photo-immunotherapy in KPC mice xenografts close to 200 mm^3^, immunocompetent mice subcutaneously implanted with KPC tumors were untreated or treated with 18 mg/kg IRI-PoP liposomes (2 mg/kg PoP dose) followed by laser irradiation on tumor using 665 nm laser with fluence rate of 250 mW/cm^2^ with fluence of 250 J/cm^2^. Mice that received ICB were intraperitoneally administered with 5 mg/kg anti-PD-1 mAb and 5 mg/kg anti-CTLA4 mAb three times at an interval of 3 days starting from the same day as the day of other treatments. It was observed that the mice receiving combined chemo-photo-immunotherapy showed complete tumor remission by 15th day [Fig. 3(a)]. Comparatively, mice that received CPT showed tumor reduction until the 12th day but grew back later rapidly. The mice that received only ICB treatment showed rapid tumor growth after 3rd day from treatment and did not show regression later. The control mice showed fastest tumor growth and had to be euthanized by the 18th day [Fig. 3(b)]. The mice receiving ICB treatment or chemophototherapy alone reached end point and had to be sacrificed respectively by 42nd day and 48th day from treatment. Conversely, mice treated with the chemo-photo-immunotherapy combination showed complete tumor remission and survived over 100 days from the treatment with no tumor recurrence. A characteristic of PDAC is desmoplasia, which can create a dense impenetrable TME containing excessive accumulation of collagen-rich extracellular matrix by stromal fibroblasts.^58–60^ This hinders the deposition of anti-cancer drugs in tumors making it almost impossible to kill pancreatic cancer cells.^61^ PDT is known to cause disruption of tumor vasculature thereby allowing enhanced tumoral drug uptake that aids in improved cell killing. Also, desmoplasia associated therapeutic resistance can be palliated by collagen photomodulation using PDT.^62^ In this way, PDT enables elevated localized tumoral drug deposition leading to faster and improved anti-cancer therapy in PDAC. However, PDT or chemotherapy, alone or in combination, did not completely cause tumor regression in PDAC (Fig. 1), possibly owing to contributions of the tumor immune-microenvironment. T cell exhaustion and dysfunction of CD8^+^ T cells leads to suppressed immune responses to cancer cells aiding in rapid cancer recurrence. To prevent this, ICB was also administered into one of the mice cohorts that received chemophototherapy. The mice that received ICB with chemophototherapy demonstrated substantially improved therapeutic results with no tumor recurrence over 100 days of the study.

**FIG. 4.**
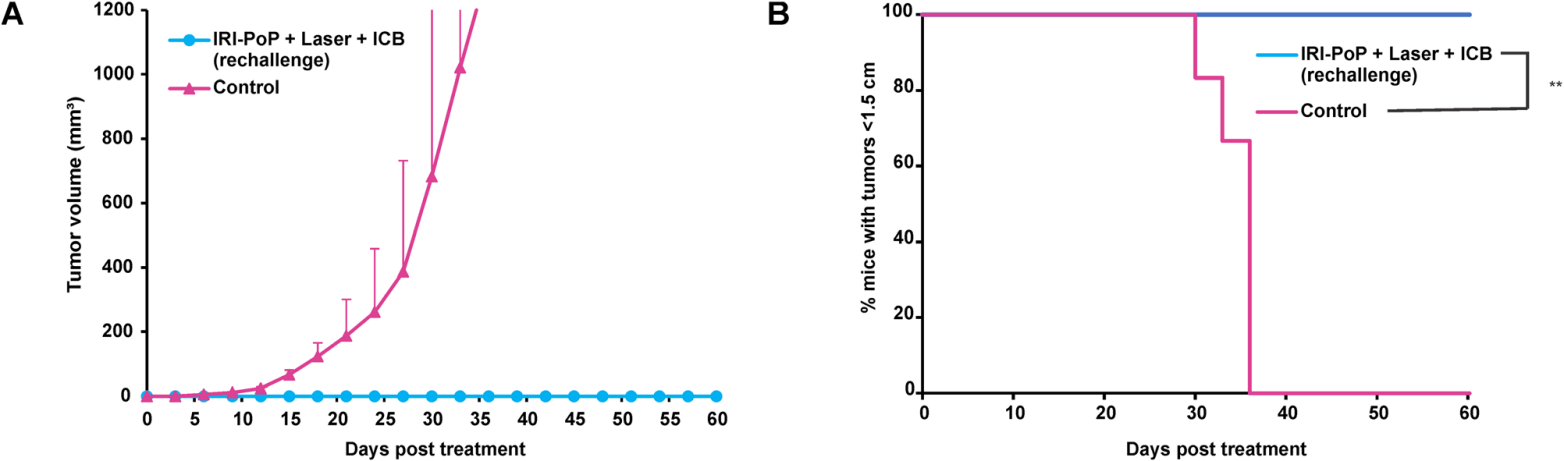
Immune response in KPC mice by CPT using IRI-PoP liposomes with ICB prevents tumor recurrence when re-challenged with tumor cells. Immunocompetent mice with KPC tumor xenografts (∼200 mm^3^) were untreated/treated with IRI-PoP liposomes with 18 mg/kg IRI dose (2 mg/kg PoP dose). Mice that received laser treatment were treated using a 665 nm laser with a laser fluence rate of 250 mW/cm^2^ with a total fluence of 250 J/cm^2^ at 1 h DLI. Mice receiving ICB (5 mg/kg anti-PD-1 and 5 mg/kg anti-CTLA4) were administered intraperitoneally three times starting from the day of laser treatment at an interval of 3 days. The cured mice were rechallenged with KPC cells to find if immune response generated by treatment could prevent tumor recurrence. (a) Tumor volume growth and (b) %mice tumors <1200 mm^3^ in volume over time. Mean ± SD for n = 6. Statistical analysis of Kaplan Meier curve was performed by Log-rank (Mantel-Cox) test, ^*^P <0.05, ^**^P <0.01.

To explore whether there was an immunological memory response caused by photodynamic chemo-immunotherapy, cured or untreated mice were re-challenged with subcutaneous inoculation of KPC cells (Fig. 4). All mice from control group showed tumor growth whereas no recurrence of tumors was observed in the previously cured mice. This indicates effective induction of immune memory response against KPC tumors in mice that received IRI-PoP + laser + ICB.

### CPT enables enhanced penetration of administered ICB antibodies in tumors

**FIG. 5.**
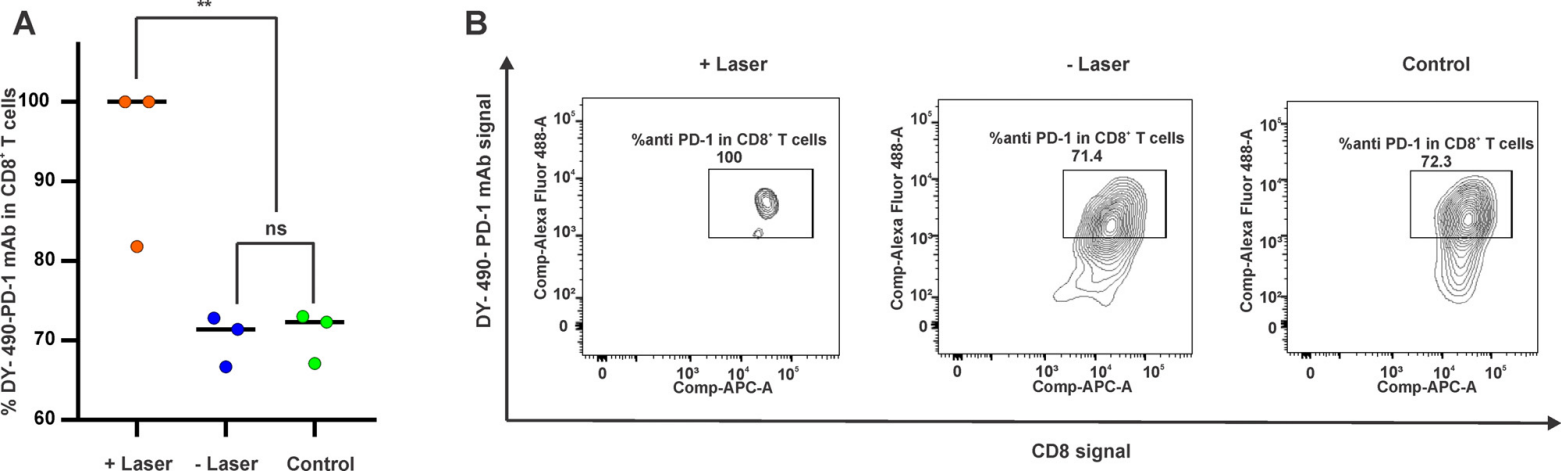
Binding of labeled anti-PD-1 to CD8^+^ T cells in KPC tumors. Mice bearing KPC tumor xenografts were either untreated or treated with 18 mg/kg IRI (2 mg/kg PoP) IRI-PoP liposomes. Mice that received laser treatment were treated using a 665 nm laser with a laser fluence rate of 250 mW/cm^2^ with a total fluence of 250 J/cm^2^ at 1 h DLI. All mice received DY490 labeled ICB (5 mg/kg anti-PD-1) intraperitoneally 1 h after laser irradiation. Mice were sacrificed after 24 h post-PD-1 administration and tumors were harvested for flow cytometry study. (a) Percentage and (b) representative flow cytometry gating of labeled PD-1 monoclonal antibody binding to CD8^+^ T cells in the KPC TME. Statistical analysis of %DY-490-mPD-1 in CD8^+^ T cells between indicated groups was performed by one-way ANOVA with Tukey, ^*^P <0.05, ^**^P <0.01. Mean ± SD for n = 3 mice per group.

To gain insight into how CPT with ICB cured medium-sized KPC tumors, mice implanted with KPC tumors were untreated or treated intravenously with 18 mg/kg IRI-PoP liposomes (2 mg/kg PoP dose) and one group was treated with a 665 nm laser with a laser fluence rate of 250 mW/cm^2^ with a total fluence of 250 J/cm^2^ at 1 h DLI. All mice were intraperitoneally administered with 5 mg/kg fluorescently labeled anti-PD-1 mAb 1 h after laser administration. All mice were sacrificed 24 h after PD-1 administration, and tumors were harvested for flow cytometry. As shown in Fig. 5(a), based on flow cytometry gating, close to 100% of CD8^+^ T cells were found to be labeled with anti-PD-1 mAb in tumors that received CPT, which was higher than other control groups (71.4% in the no laser group and 72.3% in the untreated group). This is probably due to the enhanced vascular permeability caused by disruption of tumor vasculature allowing better deposition of antibodies into the tumor. As shown in Fig. 5(b), the CPT-treated tumor contained less CD8^+^ T cells, possibly due to nonspecific CD8^+^ T cell killing by the CPT treatment itself or by the high level of IRI within the tumor. Within the three groups, the overall proportion of CD8^+^ T cells within the tumor relative to the total number of cells was similar (∼1%–2%; Fig. S4).

### Microdistribution of IRI, PoP and CD8^+^ T cells labeled with fluorescently labeled anti-PD-1

**FIG. 6.**
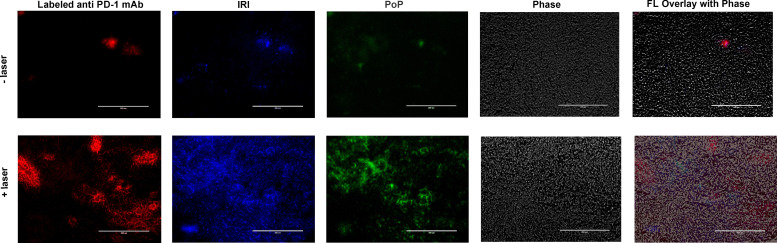
Chemophototherapy enhances the uptake of an anti-PD-1 mAb in KPC tumors with heterogenous microdistribution. KPC tumor-bearing mice were treated with IRI-PoP liposomes (18 mg/kg IRI, 2 mg/kg PoP). Mice that received laser treatment were treated using a 665 nm laser with a laser fluence rate of 250 mW/cm^2^ with a total fluence of 250 J/cm^2^ at 1 h DLI. All mice received Dy490 labeled mAb (5 mg/kg anti-PD-1) intraperitoneally after an hour from laser irradiation. After 24 h from drug administration, all mice were sacrificed and tumors were harvested. The tumors were flash-frozen and sliced using cryostat. The tumor slices were then imaged using fluorescence microscopy. Scale bar, 200 *μ*m. Representative images for n = 3 per group.

To interrogate how CPT affects the uptake and microdistribution of fluorescently labeled anti-PD-1, IRI, and PoP in tumors, mice with KPC tumors were intravenously injected with 18 mg/kg IRI dose IRI-PoP liposomes (2 mg/kg PoP dose) and then were treated with a laser using a 665 nm laser with a laser fluence rate of 250 mW/cm^2^ with a total fluence of 250 J/cm^2^ at 1 h DLI. All mice received Dy490 labeled mAb (5 mg/kg anti-PD-1) intraperitoneally after 1 h after laser irradiation. 24 h after PD-1 administration, mice were sacrificed, and tumors were harvested, flash-frozen, and cryo-sectioned. The tumor slices were then imaged using fluorescence microscopy. The figure shows the enhanced penetration of DY490 labeled anti-PD-1 mAb, IRI, and PoP in tumors that were treated with laser, indicated by red, blue, and green colors, respectively (Fig. 6). This demonstrates that CPT enables enhanced penetration of the chemotherapy drug IRI, the PoP photosensitizer responsible for PDT, as well as the ICB antibody itself. Distribution of the PD-1 antibody was heterogenous and appeared to be localized in specific parts of the tumor (also shown in another mouse tumor in Fig. S5), potentially due to extravasation from only certain active and permeabilized blood vessels following PDT.

### CPT of large established KPC tumors using IRI-PoP liposomes with ICB

**FIG. 7.**
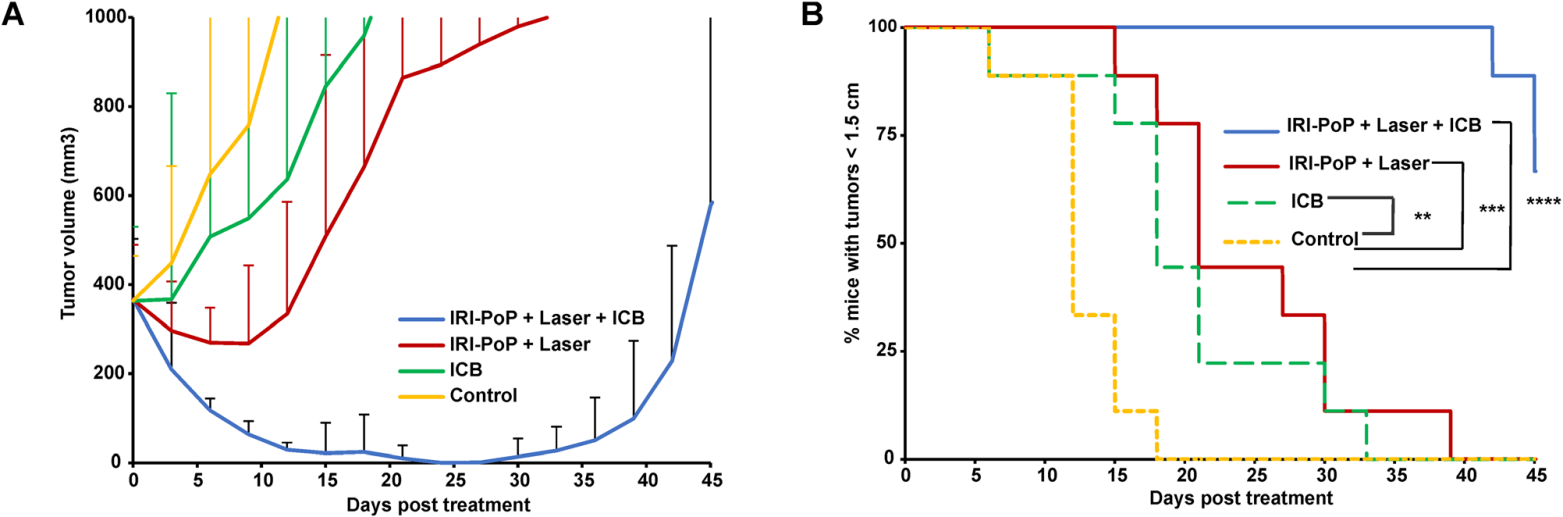
Synergistic chemo-photo-immunotherapy for big KPC tumor xenografts. Immunocompetent mice with KPC tumor xenografts (∼400 mm^3^) were untreated/treated with IRI-PoP liposomes with 18 mg/kg IRI dose (2 mg/kg PoP dose). Mice that received laser treatment were treated using a 665 nm laser with a laser fluence rate of 250 mW/cm^2^ with a total fluence of 250 J/cm^2^ at 1 h DLI. Mice receiving ICB (5 mg/kg anti-PD-1 and 5 mg/kg anti-CTLA4) were administered intraperitoneally three times starting from the day of laser treatment at an interval of 3 days. (a) Tumor volume growth and (b) %mice tumors <1.5 cm in diameter over time. Mean ± SD for n = 9. Statistical analysis of Kaplan Meier curve was performed by Log-rank (Mantel-Cox) test, ^*^P <0.05, ^**^P <0.01, ^***^P <0.001, ^****^P <0.0001.

In general, immunotherapies struggle to demonstrate tumor control in large established preclinical tumor models. To investigate the efficacy of chemo-photo-immunotherapy in large KPC mice xenografts, immunocompetent mice bearing big KPC tumors (∼400 mm^3^) were untreated or treated with 18 mg/kg IRI-PoP liposomes (2 mg/kg PoP dose) followed by laser irradiation on tumor using 665 nm laser with fluence rate of 250 mW/cm^2^ with fluence of 250 J/cm^2^. Mice that received ICB were intraperitoneally administered with 5 mg/kg anti-PD-1 mAb and 5 mg/kg anti-CTLA4 mAb three times at an interval of 3 days starting from the same day as the day of other treatments. It was observed that mice that received only chemophototherapy with IRI-PoP liposomes and laser treatments showed tumor shrinkage for about 9 days from the treatment but tumors grew rapidly after 10th day and all mice gradually reached end point of the study and had to be sacrificed [Fig. 7(a)]. The mice that received ICB continued having a similar average tumor volume for about 3 days before growing back uncontrollably and had to be sacrificed soon. The untreated mice with big tumors grew profusely reaching a mean volume of ∼1000 mm^3^ in about 15 days from the treatment and all mice had to be sacrificed by 18th day from the treatment [Fig. 7(b)]. The mice that received photodynamic chemo-immunotherapy showed distinctly improved tumor shrinkage over time as compared to other control groups. Tumors showed gradual shrinkage until about 25th day from treatment before showing traces of palpable tumor growth in some mice from 30th day which slowly grew over time. Nonetheless, this combined modality showed significantly heightened response in tumoral reduction as compared to other therapeutic measures used in this study.

## DISCUSSION

Herein, we report a combination therapy involving CPT and ICB-assisted immunotherapy in a PaCa mouse model. The growth of small KPC tumors in immunocompetent mice of ∼100 mm^3^ could be delayed but not cured by single treatment photodynamic IRI-PoP liposomes. In previous studies, while PDT alone with PoP liposomes was not effective, CPT with IRI-PoP liposomes could cure similarly sized MIA PaCa-2 tumors in immunocompromised mice.^42^ The faster growing and more aggressive nature of syngeneic tumors could contribute to the lack of KPC tumor cure with CPT alone. To investigate this, T cell exhaustion study conducted using flow cytometry demonstrated heightened expression of PD-1 in tumor-infiltrating T lymphocytes indicating dysfunction and exhaustion of T cells. To counter this, administration of ICB (anti-PD-1 and anti-CTLA-4) along with CPT in medium KPC tumors not only enabled complete regression of tumors but also stayed cancer free for over 100 days of the study. Furthermore, rechallenging the cured mice with subcutaneous KPC cells implantation resulted in tumor rejection without regrowth.

PDT has been long been established to enhance the delivery of liposomes to tumors.^63^ Indeed, we have shown that IRI-PoP benefits from PDT-induced tumor vessel permeabilization, leading to enhanced drug deposition following tumor irradiation.^42^ Flow cytometry studies with fluorescently (DY490) labeled anti-PD-1 showed enhanced mAb deposition in mice that received laser treatment, which presumably allowed effective immune response against cancer cells. Microdistribution analysis to inspect the distribution of drugs and ICB in tumor using fluorescence microscopy also illustrated the improved deposition of drugs and ICB in tumors that received laser therapy as compared to no laser treatment. Apart from the efficient elimination of cancer cells contributed jointly by photodynamic effect and the augmented tumoral uptake of chemotherapy drug IRI into the cells, the boosted deposition of ICB might have acted cohesively in developing a strong immune response by activating the negative feedback loop that impedes the dysfunction of activated immune response. Another study to investigate the impact of this combination therapy on big KPC tumors (∼400 mm^3^) showed faster tumor shrinkage initially in mice that received CPT with ICB as compared to other control groups, but later started showing tumor relapse that gradually grew bigger.

Some limitations of this study should be noted. We only assessed cellular responses based on tumor infiltrating T cells, whereas it is known that a wide variety of other cells are involved in the PDT adaptive immune response, including neutrophils.^64^ Furthermore, induction of immunogenic cell death was not assessed, which is known to lead to enhanced anti-tumor immune responses.^65^ Future studies should aim to assess the dosimetry of the various components of this treatment, including the type and timing of ICB antibody, the dose of IRI-PoP, and the fluence and DLI of laser treatment.

## CONCLUSION

This study points to the potential of the combination of CPT and immunotherapies in not only curing medium-sized established syngeneic tumors based on leveraging of cancer immune responses but also effectively shrinking large established tumors to a point which they could be resected in a surgical setting. The KPC immunocompetent murine tumor model benefits from combining ICB with CPT.

## METHODS

### Materials

Porphyrin–phospholipid (PoP) was prepared as described.^51^ 1,2-Distearoyl-sn-glycero-3-phosphocholine (DSPC, Corden Pharma CAT# LP-R4-076), cholesterol (PhytoChol, Wilshire Technologies Inc.), 1,2-distearoyl-sn-glycero-3-phosphoethanolamine-N-[methoxy(polyethylene glycol)-2000] (MPEG-2000-DSPE, Corden Pharma CAT# LP-R4-039), and IRI (LC Laboratories CAT# I-4122) were used for PoP liposomes preparation. Anti-CTLA-4 (Clone: 9H10, CAT# BP0131), Anti-PD-1 (Clone: RMP1-14, CAT# BP0146) were obtained from BioXCell.

### Liposome preparation and characterization

IRI-PoP liposomes were prepared as recently described.^42^ To prepare a 5 ml batch (20 mg/ml lipids), liposomes were prepared by slowly injecting 1 ml ethanol at 60 °C into powdered lipids (DSPC: Chol: PoP: MPEG-2000-DSPE in the molar ratio of 58.7:40:1:0.3), followed by injecting 4 ml of 120 mM of ammonium sucrose octasulfate (Toronto Research Chemicals, CAT# S698990) at 60 °C. Then the lipid mixture was carefully passed 10 times through a nitrogen-pressurized extruder (Northern lipids) having sequentially stacked polycarbonate membranes of 0.2, 0.1, and 0.08 *μ*m pore size. After extrusion, the liposomes were dialyzed against 800 ml solution of 145 mM sodium chloride with 5 mM HEPES (pH 6.5) with at least two changes of buffer. To load IRI in the liposomes, the drug was incubated with liposomes at a drug: lipid molar ratio of 1:8 for 1 h at 60 °C. The drug loading efficiency of the liposomes was determined by running 100 *μ*l of the liposomes diluted in 1 ml phosphate buffered saline (PBS) over a Sephadex G-75 column and 24 column fractions were collected. The fluorescence of IRI and PoP in the column fractions was measured using a TECAN plate reader, and the loading efficiency was calculated as the percentage of the drugs that co-eluted with the liposomes. IRI was measured using fluorescence with an excitation of 370 nm and an emission of 435 nm, and PoP was measured using fluorescence with an excitation of 420 nm and an emission of 670 nm. Light-triggered IRI release was measured in 20% bovine serum at 37 °C in a fluorometer (PTI) using a 665 nm laser diode (RPMC laser, LDX-3115–665) and %Release was determined by the formula: %Release= (F_final_ − F_initial_)/(F_X-100_ − F_initial_) × 100%. Liposome size was ∼100 nm, as measured by dynamic light scattering (Nanobrook 90 plus PALS). Cryo-electron microscopy was carried out as previously described.^42^

### Tumor growth inhibition

All murine studies were performed according to the protocols approved by the University at Buffalo's Institute of Animal Care and Use Committee (IACUC, protocol # BME03112Y). Cells isolated from a mouse with a spontaneous KrasLSL-G12D/+/Trp53LSL-R172H/+/Pdx-1-Cre tumor (KPC cells) were kindly provided by Dr. Huan Meng from the University of California, Los Angeles.^52^ KPC cells were cultured in Dulbecco's Modified Eagle's Medium High Glucose Sodium Pyruvate (Gibco 11995-065) supplemented with 10% fetal bovine serum, 1% antibiotics, and plasmocin (5 *μ*g/ml). Immunocompetent B6129SF1/J six-week female mice (Jackson Laboratory CAT# 101043) were inoculated subcutaneously with 2 × 10^6^ KPC pancreatic ductal adenocarcinoma cells. When tumors reached the indicated tumor volume required for the study, mice were randomly grouped into the required cohorts: control, IRI-PoP + laser, IRI-PoP − laser, empty PoP liposomes + laser (Fig. 1); control, ICB, IRI-PoP + laser + ICB, IRI-PoP + laser (Figs. 3 and 7). To determine the anti-tumor efficacy of IRI-PoP liposomes in tumor inhibition of KPC tumors, the mice were untreated or treated with 18 mg/kg IRI-PoP liposomes or empty PoP liposomes with an equivalent PoP dose of 2 mg/kg. The mice that received laser treatment were treated using a 665 nm laser with a fluence rate of 250 mW/cm^2^ at a total fluence of 250 J/cm^2^ at 1 h drug-light-interval (DLI). The mice that received ICB treatment were intraperitoneally injected with 5 mg/kg anti-PD-1 mAb and 5 mg/kg anti-CTLA4 mAb three times at an interval of 3 days. To re-challenge cured mice, cured or untreated mice were subcutaneously implanted with KPC cells and monitored to find tumor growth in them. Mice health was monitored throughout the study. Tumor volume was calculated using the ellipsoid formula: volume = π/6 ×  L × W^2^, where L and W are length and width of the tumor, respectively. All mice were sacrificed when the tumors reached 1.5 cm in diameter or if ulceration occurred.

### Antibody staining

For labeling anti-PD-1, dialysis of antibody (1 mg/ml) was done into 50 mM bicarbonate buffer, pH 9.0 for two times followed by incubating the antibody with DY-490 (5:1 molar ratio) in the dark for 1–2 h. The labeled Ab was then dialyzed in PBS at 4 °C at least three times. DY-490 labeled anti-PD-1 mAb was later administered in mice for flow cytometry studies and microdistribution studies using fluorescence microscopy. For T-cell exhaustion analysis, tumor cells prepared as described below were stained with live/dead fixable dye (Invitrogen; CAT#: L34857, 500 × diluted), Fc-block (BD, CAT#: 553142, 100 × diluted), and the antibody against CD16/CD32 (100 × diluted), CD45-AF700 (Biolegend, CAT#: 103127, 200 ×  diluted), APC-CD8a antibody (Biolegend, CAT#: 100712, 200 ×diluted), and PD-1-FITC (Biolegend, CAT#: 135213, 200  ×  diluted). For detecting labeled PD-1 antibody binding to CD8^+^ T cells, cells were stained with live/dead fixable dye, Fc-block, and the antibody against CD45 (200  ×  diluted) and CD8a (200  ×  diluted). Cells were incubated with the mixture of mentioned antibodies for 30 min at 4 °C and then washed twice before the flow cytometry analysis.

### Tumor cell studies

Immunocompetent mice bearing KPC tumor xenografts were injected with IRI-PoP liposomes with 18 mg/kg IRI (2 mg/kg PoP) followed by laser irradiation with a 665 nm laser with fluence rate of 250 mW/cm^2^ at fluence of 250 J/cm^2^. The mice were administered intraperitoneally with fluorescently labeled 5 mg/kg anti-PD-1 mAb. After 24 h from drug administration, the mice were sacrificed, and spleens/tumors were harvested. For tumor-infiltrating lymphocyte (TIL) studies, freshly collected tumors were washed in PBS and cut into 1–2 mm pieces. The tiny tumor pieces were then digested with collagenase type I (2 mg ml^−1^) and DNase I (100 *μ*g ml^−1^) for 1 h at 37 °C in the cell culture incubator. The digested tumor tissue was then dissociated by passing it through a 70 *μ*m strainer with a sterile 3 ml syringe plunger, and cells were collected. The cells were then washed with cold PBS and used in further experiments. For splenocytes preparation, freshly harvested spleens were dissociated and filtered through a 70 *μ*m cell strainer with a sterile 3 ml syringe plunger. The dissociated tissue was then washed with 5 ml of cold PBS, and the cells were collected into a 50 ml tube. Then, the cells were centrifuged at 500 × *g* for 5 min. The supernatants were carefully discarded keeping the pellet intact. Furthermore, red blood cells in the pellet were lysed by incubating the pellet in 5 ml of red blood cell lysis buffer for 5 min. After 5 min of incubation, 35 ml PBS was added to the cell suspension followed by centrifugation, and cell pellets were collected for further experiments. Antibodies were stained as mentioned above. For flow cytometry studies, a BD LSRFortessa X-20 cytometer was used, and data analysis was done using Flowjo (version 10) software.

### Fluorescence microscopy

For imaging cellular uptake, KPC cells were seeded in six-well plates at a density of 1 × 10^4^ cells in 200 *μ*l of Dulbecco's Modified Eagle Medium (DMEM) with 10 % fetal bovine serum (FBS), 1% penicillin/streptomycin (P/S), 2 mM L-glutamate, and 5 *μ*g/ml plasmocin. The attached cells were incubated for 4 h with IRI-PoP liposomes with IRI concentration of 10 *μ*g/ml with liposomes that were or were not pretreated with laser treatment. To trigger IRI release, IRI-PoP liposomes were diluted to 100 *μ*g/ml in medium and irradiated using 665 nm laser diode at 300 mW/cm^2^ for 3 min and further diluted to 10 *μ*g/ml when incubated with KPC cells. Later, the cells were washed with PBS twice, followed by incubation with 1000-time diluted SYTO™ 9 Green Fluorescent Nucleic Acid Stain (5 *μ*M) for 20 min. Samples were imaged with a Leica Stellaris 5 Confocal Microscope using a 10× objective lens.

For imaging the tumor microenvironment, immunocompetent mice bearing KPC tumor xenografts were injected with IRI-PoP liposomes with 18 mg/kg IRI dose (∼2 mg/kg PoP dose) followed by laser irradiation with a 665 nm laser with fluence rate of 250 mW/cm^2^ at fluence of 250 J/cm^2^. All mice were administered intraperitoneally with fluorescently labeled 5 mg/kg anti-PD-1 mAb post-1 h from laser treatment. 24 h after drug administration, mice were sacrificed and tumors were harvested. The tumors were quickly embedded in optimal cutting temperature (OCT) mounting medium and immediately snap-frozen using liquid nitrogen and stored in −80 °C refrigerator. The frozen tumors were thinly sliced (12 *μ*m) using a Cryostat (H/I Bright OTF5000). The tumor slices were mounted on glass slides and stored at −20 °C. The tumors were imaged with a fluorescence microscope (EVOS FL Auto) using a DAPI filter cube (357 nm excitation; 477 nm emission) for IRI, and a custom filter cube (400 nm excitation; 679 nm emission) for PoP and DY490 labeled anti-PD-1 mAb was imaged with filter cubes with 470 nm excitation and 593 nm emission.

### *In vitro* cytoxicity

KPC cells were seeded in 96-well plates at a density of 1 × 10^4^ cells in 200 *μ*l of DMEM with 10 % FBS, 1% penicillin/streptomycin, 2 mM L-glutamate, and 5 *μ*g/ml plasmocin. The attached cells were treated with IRI-PoP liposomes with a series of irinotecan concentration at 100, 10, 1, 0.1, 0.01, and 0.001 *μ*g/ml IRI with or without prior laser treatment to induce full IRI release (IRI-PoP liposomes were diluted to 100 *μ*g/ml in medium and irradiated using 665 nm laser diode at 300 mW/cm^2^ for 3 min). Cells were incubated with the drugs at the indicated concentrations for 72 h. All measurements were done in triplicate. Cell viability was measured with the Alamar Blue assay.

### Statistical analysis

Statistical analysis of data were performed using GraphPad Prism software Version 8 using methods indicated in the figure captions.

## SUPPLEMENTARY MATERIAL

See the supplementary material file includes data on liposome characterization as well as uptake and cytotoxicity in KPC cells.

## Data Availability

The data that support the findings of this study are available from the corresponding author upon reasonable request.
